# Supplementary material for: Association of HOMA-IR Versus TyG Index with Diabetes in Individuals Without Underweight or Obesity
Source: Healthcare (Basel). 2024 Dec 5;12(23):2458. doi: 10.3390/healthcare12232458 (PMC11641693; doi:10.3390/healthcare12232458)
Supplement: Supplementary file 1 [file healthcare-12-02458-s001.zip › healthcare-3310764-supplementary.pdf]

Association of HOMA-IR Versus TyG Index with Diabetes in Individuals Without Underweight or Obesity

Supplementary Table S1. Characteristics of non-parametric variables among study subjects.

	Overall subjects (n = 10471)	Male subjects (n = 4298)	Female subjects (n = 6173)	*P* Value
Age, year†	54.0 (40.0, 66.0)	54.0 (38.0, 66.0)	53.0 (40.0, 66.0)	0.544
TyG index†	8.4 (8.0, 8.8)	8.5 (8.1, 9.0)	8.4 (8.0, 8.8)	0.001
Height, cm†	162.0 (156.3, 168.8)	165.9 (159.6, 172.1)	159.8 (154.9, 165.5)	0.001
Body mass, kg†	58.0 (52.9, 64.3)	61.5 (55.3, 67.8)	56.2 (51.8, 61.4)	0.001
Glucose, mg/dL†	94.0 (89.0, 102.0)	96.0 (90.0, 104.0)	94.0 (88.0, 101.0)	0.001
SBP, mm Hg†	117.7 (107.3, 130.0)	119.3 (109.3, 130.7)	116.0 (106.0, 130.0)	0.001
DBP, mm Hg†	74.0 (68.7, 81.3)	75.3 (69.3, 82.7)	73.3 (68.0, 80.3)	0.001

Values are median and inter-quartile range. †Mann-Whitney U test was applied to assess the difference between groups. DBP = diastolic blood pressure; SBP = Systolic blood pressure

Supplementary Table S2. Characteristics of study subjects.

	Overall subjects (n = 10471)			Male subjects (n = 4298)			Female subjects (n = 6178)			P Value
Total cholesterol, mg/dL†	189.9	±	37.4	187.7	±	37.9	191.5	±	36.9	0.001
Creatinine, mg/dL†	0.80	±	0.32	0.86	±	0.34	0.76	±	0.30	0.001
AST, U/L†	23.3	±	13.5	24.2	±	14.9	22.7	±	12.3	0.001
ALT, U/L†	19.7	±	14.3	21.2	±	15.4	18.7	±	13.4	0.001
Recreational MVPA, min†	62.6	±	149.0	87.2	±	177.8	45.4	±	122.2	0.001
Total energy intake, kcal/d†	1855.7	±	837.0	2002.2	±	930.0	1753.6	±	748.8	0.001
Protein, g/d†	68.0	±	42.4	73.0	±	51.7	64.5	±	34.1	0.001
Fat, g/d†	44.7	±	34.7	47.4	±	38.2	42.8	±	32.0	0.001
Carbohydrate, g/d†	276.7	±	113.5	291.6	±	118.0	266.3	±	109.0	0.001
Houshold income (%)										0.001
Low	1962 (18.7)			733 (17.1)			1229 (19.9)			
Lower-middle	2536 (24.2)			1018 (23.7)			1518 (24.6)			
Upper middle	2877 (27.5)			1189 (27.7)			1688 (27.3)			
High	3096 (29.6)			1358 (31.6)			1738 (28.2)			
Education level (%)										0.001
Primary school	2110 (20.2)			616 (14.3)			1494 (24.2)			
Middle school	1004 (9.6)			406 (9.4)			598 (9.7)			
High school	3377 (32.3)			1520 (35.4)			1857 (30.1)			
College	3980 (38.0)			1756 (40.9)			2224 (36.0)			
Drinking (%)										0.001
Never	3130 (29.9)			851 (19.8)			2279 (36.9)			
≤ once a week	5356 (51.2)			2086 (48.5)			3270 (53.0)			
2-3 times/week	1401 (13.4)			886 (20.6)			515 (8.3)			
≥ 4 times/week	584 (5.6)			475 (11.1)			109 (1.8)			
Smoking (%)										0.001
Never	6524 (62.3)			1049 (24.4)			5475 (88.7)			
Former smoking	2377 (22.7)			1964 (45.7)			413 (6.7)			
Current smoking	1570 (15.0)			1285 (29.9)			285 (4.6)			

Values are means ± SD. †Mann-Whitney U test was applied to assess the difference between groups. AST = aspartate transaminase; ALT = alanine aminotransferase; MVPA = moderate-to-vigorous physical activity

Supplementary Table S3. Sex-specific characteristics of biochemical parameters and lifestyle and trends by TyG Index tertiles.

	A. MHNW (95% CI)		B. Pre-MONW (95% CI)		C. MONW (95% CI)		Post-hoc	SS‡	trend‡
Male subjects (n = 4298)	n = 804		n = 2305		n = 1189				
AST, U/L†	22.1 ± 9.8	(21.4, 22.8)	24.3 ± 14.9	(23.6, 24.9)	25.6 ± 17.5	(24.6, 26.6)	A < B < C	7.74	0.001
ALT, U/L†	17.9 ± 12.1	(17.1, 18.8)	21.2 ± 15.8	(20.5, 21.8)	23.5 ± 16.3	(22.6, 24.5)	A < B < C	11.97	0.001
Total cholesterol, mg/dL†	187.7 ± 33.2	(185.4, 190.0)	187.8 ± 37.9	(186.2, 189.3)	187.5 ± 40.9	(185.2, 189.8)	NS	-0.34	0.733
Creatinine, mg/dL	0.83 ± 0.51	(0.80, 0.87)	0.86 ± 0.29	(0.84, 0.87)	0.89 ± 0.26	(0.87, 0.90)	A, B < C	7.06	0.001
Recreational MVPA, min	102.0 ± 182.3	(89.3, 114.6)	85.5 ± 177.1	(78.3, 92.7)	80.7 ± 175.7	(70.7, 90.7)	A > C	-4.57	0.001
Total energy intake, kcal/d	2023.4 ± 961.4	(1956.8, 2089.9)	2006.3 ± 948.8	(1967.6, 2045.1)	1980.0 ± 870.1	(1930.5, 2029.5)	NS	-0.45	0.655
Protein, g/d	75.0 ± 42.1	(72.1, 78.0)	73.4 ± 59.9	(70.9, 75.8)	71.0 ± 38.7	(68.8, 73.2)	A > B > C	-2.00	0.05
Fat, g/d†	52.3 ± 39.3	(49.5, 55.0)	47.9 ± 40.3	(46.2, 49.5)	43.0 ± 32.4	(41.2, 44.9)	A > B > C	-6.06	0.001
Carbohydrate, g/d	281.5 ± 117.5	(273.4, 289.6)	292.5 ± 115.8	(287.8, 297.2)	296.8 ± 122.4	(289.8, 303.8)	A < C	2.77	0.01
Female subjects (n = 6173)	n = 1477		n = 2932		n = 1764				
AST, U/L†	21.0 ± 8.0	(20.6, 21.4)	22.7 ± 13.8	(22.2, 23.2)	24.1 ± 12.6	(23.5, 24.7)	A < B < C	9.85	0.001
ALT, U/L†	16.3 ± 14.9	(15.6, 17.1)	18.5 ± 11.2	(18.1, 18.9)	21.1 ± 14.9	(20.4, 21.8)	A < B < C	14.43	0.001
Total cholesterol, mg/dL†	193.3 ± 32.5	(191.6, 195.0)	191.8 ± 37.2	(190.4, 193.1)	189.5 ± 39.8	(187.6, 191.3)	A > C	-3.20	0.01
Creatinine, mg/dL†	0.74 ± 0.21	(0.73, 0.75)	0.76 ± 0.27	(0.75, 0.77)	0.80 ± 0.38	(0.78, 0.81)	A, B < C	6.67	0.001
Recreational MVPA, min†	48.0 ± 125.2	(41.6, 54.4)	47.7 ± 127.7	(43.0, 52.3)	39.4 ± 109.6	(34.3, 44.6)	NS	-2.42	0.05
Total energy intake, kcal/d†	1815.0 ± 795.5	(1774.4, 1855.6)	1717.7 ± 720.2	(1691.6, 1743.7)	1761.9 ± 752.0	(1726.8, 1797.1)	A > B, B <C	-1.14	0.253
Protein, g/d†	68.0 ± 35.9	(66.2, 69.9)	63.0 ± 32.9	(61.9, 64.2)	64.0 ± 34.5	(62.4, 65.7)	A > B, C	-3.04	0.01

Fat, g/d†	47.7	±	33.7	(46.0, 49.4)	41.7	±	30.7	(40.6, 42.8)	40.7	±	32.1	(39.2, 42.2)	A > B > C	-7.77	0.001
Carbohydrate, g/d	266.7	±	114.2	(260.8, 272.5)	261.3	±	106.2	(257.5, 265.2)	274.3	±	108.6	(269.2, 279.3)	B < C	3.12	0.01

Values are means ± SD. †Mann-Whitney U test was applied to assess the difference between groups. ‡Jonckheere-Terpstra test was used to assess the trend among three groups. NS = not significant; AST = aspartate transaminase; ALT = alanine aminotransferase; MVPA = moderate-to-vigorous physical activity
